# Postdiverticular Stasis—Introduction of a New Etiology for Small Intestine Obstruction: A Case Series

**DOI:** 10.1155/2012/802743

**Published:** 2012-07-12

**Authors:** Seyed Abdollah Mousavi, Hassan Karami

**Affiliations:** ^1^Department of Pediatric Surgery, Faculty of Medicine, Mazandaran University of Medical Sciences, Sari, Iran; ^2^Department of Pediatric Gastroenterology, Faculty of Medicine, Mazandaran University of Medical Sciences, Sari, Iran

## Abstract

*Introduction*. Intestinal obstruction in the setting of Meckel's diverticulum in young age and with orange and meat bezoar is a rare, previously unreported condition. Since the obstruction point is located immediately after Meckel's diverticulum in our patients, we attempt to introduce “*localized peristalsis insufficiency*” as a new etiology for small intestine obstruction while reviewing the findings of previous studies. *Conclusion*. Intestinal obstruction in the setting of Meckel's diverticulum and with orange and meat bezoar is a rare, previously unreported condition. Considering the previous reports, we may present the theory of *localized peristalsis insufficiency* in patients with Meckel's diverticulum.

## 1. Introduction

Small intestine obstruction occurs due to a variety of etiologies, including Meckel's diverticulum [1]. Although intestinal obstruction due to a phytobezoar within a Meckel's diverticulum is extremely rare, it has nevertheless been noted by some researchers [2–4].

All studies published in English incriminate bezoar as the cause of mechanical obstruction, while we believe that the peristalsis of small intestine is compromised in the vicinity of the diverticulum as the tubular form of intestinal muscles is disrupted, leading to sluggish transit and stasis after the diverticulum and, eventually, obstruction. In this paper, we shall present two cases with obstructive symptoms of the small intestine in the setting of bezoar in Meckel's diverticulum, and use them to suggest a new etiology for intestinal obstruction—localized peristalsis insufficiency.

## 2. Case Reports

### 2.1. Case 1

**Figure 1:**
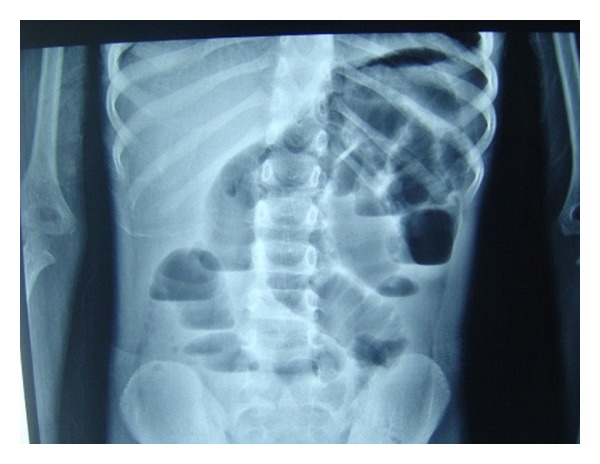
Abdominal X-Ray indicated loop distention.

**Figure 2:**
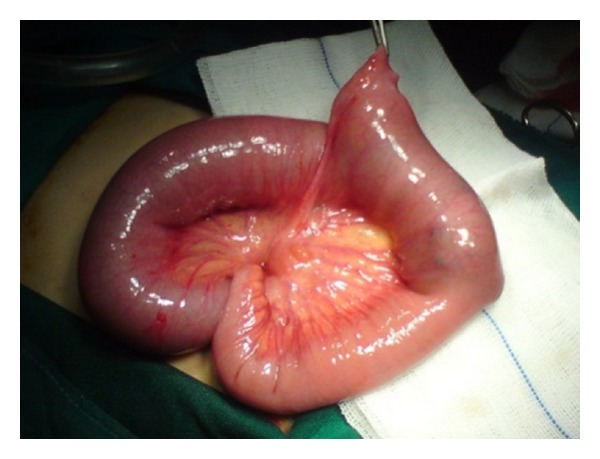
Distended intestinal loops after diverticulum—mass effect.

**Figure 3:**
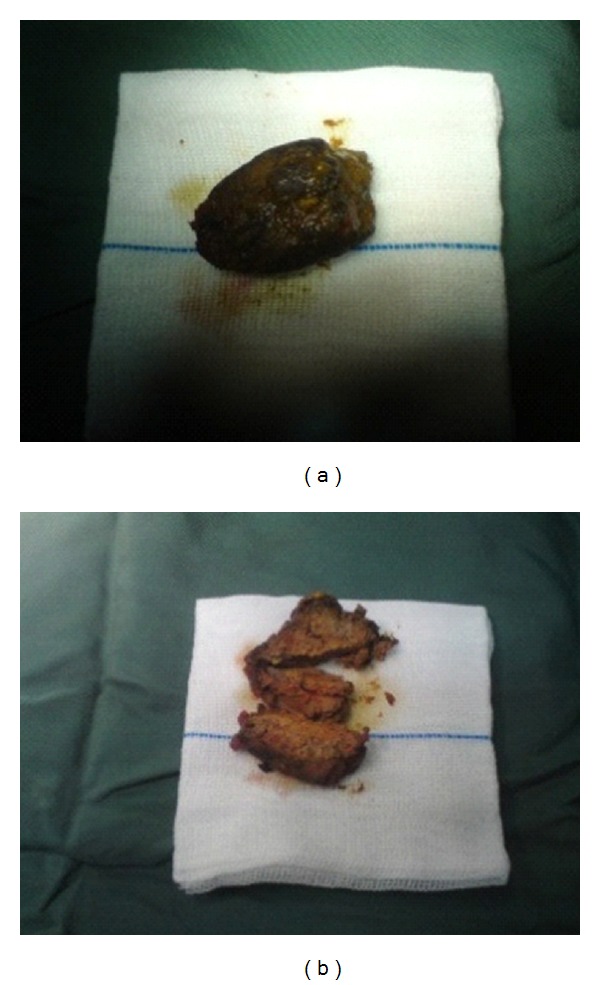
Lump of meat forming a bezoar.

A 9-year-old boy was admitted to the emergency department with abdominal pain from 3 days ago. The pain was colicky in nature and 3 episodes of biliary vomiting were reported. Simple abdominal radiography indicated loop distention in the small intestine with air-fluid levels (Figure 1). The child was submitted to laparotomy: we found severely distended intestinal loops in jejunum and ileum, with the distension proceeding as far as a Meckel's diverticulum (Figure 2). A hard mass was palpated at some 5 cm after the Meckel's diverticulum, suggesting that obstruction originated at some point after the diverticulum. Enterotomy produced a round mass measuring 4 × 5 cm and brown in color. Sectioning the mass demonstrated lumps of meat, forming bezoar (Figure 3). Histopathologic studies of the diverticulum reported ectopic gastric tissue. A history obtained after surgery indicated that the child had consumed meat a week ago.

### 2.2. Case 2

**Figure 4:**
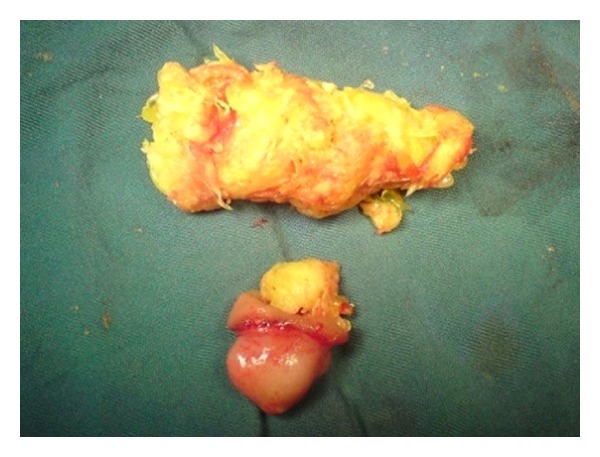
Orange impacted at the diverticulum forming a bezoar.

A 5-year-old boy referred to the pediatric gastroenterology clinic, complaining of colicky abdominal pain and 5 episodes of vomiting from yesterday. No history of surgery or disease was mentioned. Simple abdominal radiography depicted signs of intestinal obstruction. The patient was admitted to the operating room with a diagnosis of acute appendicitis. The appendix was found to be normal. Severe loop distention was observed as far as 3-4 cm after a Meckel's diverticulum. Enterotomy revealed a quantity of orange impacted at the diverticulum, extending as far as 5 cm after the diverticulum and causing obstruction at this point (Figure 4). Histopathology reported no ectopic tissue. After the surgery, the parents mentioned that the child had eaten large quantities of orange 2 days ago.

In both cases there were no preexisting motility disorders or predisposing factors. Except Meckel's diverticulum any histopathologic problems such as changes in number of ganglions or neuromotor units were not seen.

## 3. Discussion

Meckel's diverticulum is a congenital, blind pouch in the intestine, resulting from incomplete obliteration of the vitelline duct during fifth week of gestation [5]. Numerous studies have reported small intestine obstruction in the setting of Meckel's diverticulum, mostly due to the following etiologies: (a) volvulus of small intestine around a fibrous; (b) intussusceptions; (c) Littre's hernia; (d) entrapment of small bowel beneath a mesodiverticular band; (e) stricture secondary to chronic diverticulitis; (f) Meckel's diverticulum lithiasis, and some other rare causes, including tumors of diverticulum and obstruction secondary to phytobezoar formation in diverticulum [1].

A review of the literature reveals a few studies which reported bezoar as an etiology for small intestine obstruction. Bezoars of the small intestine are mostly phytobezoars [6, 7] and less frequently trichobezoar [8, 9].

A limited number of studies reported bezoar and Meckel's diverticulum together [3, 4], although none of them mention intestinal motility disorder as an etiology for obstruction.

Except a report by Duman et al. [3] who observed a phytobezoar with obstructive symptoms in a 16-month-old infant, the age of patients in other reports ranged from 44 to 78 years. In addition, the majority of diverticula were mentioned to be the result of immigration from a faulty stomach [7].

Although Tayeb et al. [6] and Balducci et al. [2] reported jejunal diverticulosis as the setting for obstruction, the latter author attributed the obstruction to inflammatory stenosis caused by repeated episodes of diverticulitis, volvulus or intussusception, and voluminous jejuna stones.

Generally, the causes of intestinal obstruction fall into one of three categories: extraluminal, intraluminal, or intrinsic to the bowel wall.

It is a fact that small intestine peristalsis is caused by contractions of the muscularis propria: contraction of the outer longitudinal muscles caused bowel shortening, while contraction of the inner circular layer is responsible for luminal narrowing. During the fasting period between meals, these cyclical contractions sweep the bowel regularly [10].

Now let us consider an intestine with diverticulum. It seems that peristalsis becomes insufficient at the level of the diverticulum as a result of the incomplete cylindrical formation of the intestine. It occurs particularly in the case of Meckel's diverticulum (a “true” diverticulum) where the contractions proceed along the detour as far as the tip of the diverticulum. Therefore, when a massive food chunk, such as a bezoar, reaches this point, it will be stopped, causing stasis and obstruction. This type of obstruction may be named “localized peristalsis insufficiency” and added to obstructive etiologies under “factors intrinsic to the bowel wall.”

Phytobezoar is a collection of plant materials, such as peels, seeds, and fibers, which cannot be digested and may rarely (2.9%) cause small intestine obstruction. In a large number of cases, it originates in the stomach following gastric surgery (vagotomy) and long-term stopping of food in the stomach. Moreover, they reported persimmon as the most common fruit [7]. We found two previously unreported types of bezoar: orange and meat. Although children of this age usually do not have problems chewing, eating in haste and insufficient mastication alongside “*localized peristalsis insufficiency*” may nevertheless lead to intestinal obstruction.

## 4. Conclusion

Intestinal obstruction in the setting of Meckel's diverticulum and with orange and meat bezoar is a rare, previously unreported condition. Considering the previous reports, we may present the theory of localized peristalsis insufficiency in patients with Meckel's diverticulum. However to prove this idea, further investigations are needed in the future.
